# Assisted resolution and self-change: differences between healthcare systems

**DOI:** 10.1192/bjo.2024.760

**Published:** 2024-10-04

**Authors:** Morten Hesse, Julie Brummer, Anette Søgaard Nielsen

**Affiliations:** Center for Alcohol and Drug Research, Aarhus University, Copenhagen, Denmark; Unit of Clinical Alcohol Research, Institute of Clinical Research, University of Southern Denmark, Odense, Denmark; and Department of Mental Health, Odense, Denmark

**Keywords:** Alcohol, recovery, treatment, self-change, mental health

## Abstract

This editorial discusses a study by Day and colleagues, in which the authors investigated the prevalence of resolution of alcohol and other drug problems in the UK and compared people who resolved their problems with and without treatment.

Day et al recently published a study on resolution of alcohol and other drug (AOD) problems based on a national survey from the UK.^1^ The authors distinguished between assisted and self-change pathways to recovery. The former characterise persons who report that they have recovered from AOD problems and have received help from formal external services to address the problematic use. The latter characterise individuals who report recovering from AOD problems and who never received such help. The authors analysed the prevalence of assisted resolution versus self-change and factors associated with each. One in 20 UK citizens reported having had substance use problems in the past, from which they had since recovered. Among those who reported a resolved substance use problem, half reported an ‘assisted’ pathway to resolution, whereas the other half reported ‘self-change’.

The survey had some important limitations that characterise a number of similar studies: the lack of a precise estimation of what an AOD problem entails, a cross-sectional design, and the absence of a precise description of what it means to have resolved a problem.^2^ However, it was also one of very few studies that have been conducted on this subject outside the USA. That alone adds some value to the study, because it is one of the few opportunities to gain some insight into results of this type of research outside North America.

This commentary will attempt to dig into the lessons learned from this and from a similar study from the USA. What can the similarities and the differences between the two studies teach us? What are the implications for practice, if any? The commentary will also consider what type of research is needed to obtain a better understanding of assisted change and self-change.

Very little research has been done on this topic outside the USA. Day et al based their methodology on a US survey study conducted in 2016, which found that more than 9% of the respondents reported resolving AOD problems.^3^ By contrast, in the UK study, only 5% reported resolving AOD problems. Seven years have passed between the US study and the UK study, and many changes have occurred globally and in each of the two countries that may explain some of the differences. For example, an opioid epidemic has caught the world's attention in the intermediary time,^4,5^ and such an epidemic may influence how people understand questions about a past problem: they may interpret their past problem as being less serious in light of news stories about thousands of overdoses, or they may be more concerned that their past use was in fact a real problem.

Nevertheless, it is interesting to think about the two studies in relation to each other. There were some informative similarities between them. For example, in both countries, around half of those who resolved an AOD problem identified as being ‘in recovery’.^1,6^ In addition, in both countries, opioid problems were more strongly associated with assisted resolution compared with alcohol problems. In both cases, mental health problems were associated with assisted resolution.

The differences between the findings in the two countries were, however, more profound. Nearly twice as many people felt that they had resolved an AOD problem in the US compared with the UK. Although some of the difference could be explained by a higher prevalence of problematic substance use in the USA, UK and US citizens consume a similar amount of alcohol on an annual basis.^7^ Thus, it does not seem plausible that the USA has double the prevalence of AOD problems compared with the UK, based on other drug use alone. This raises the question: do US and UK respondents have different understandings of the question ‘Did you use to have a problem with drugs or alcohol, but no longer do’? Are there cultural differences in the thresholds for identifying use as problematic,^8^ such that the average US respondent is more likely to think of a similar pattern of drinking or drug use as problematic, compared with the UK respondent? Is it possible that aspects of US culture that focus on self-reliant individuals overcoming difficulties through personal strength play a part?^8,9^ Maybe the average US respondent puts more emphasis on personal change history than the UK respondent? Or perhaps the more religious US respondents have a different view of AOD use based on their stronger religious values?^10^ Future work should investigate the cross-cultural validity of this type of question.

It is also worth noting some further minor differences between the findings of the two studies. In the UK, with free, universal healthcare, formal treatment was more common than self-help (35% *v.* 30%). The opposite was true in the USA, where 45% used self-help and only 28% used formal treatment – perhaps a result of less access to treatment, especially among the uninsured.^11^ These findings point to the need for more cross-national research on the impact of policy changes in terms of access to treatment for substance use problems and other types of support services.

Studies such as the present one help us to understand more about the differences between assisted resolution and self-change in different settings with different healthcare systems. But are there practical ramifications? More than 20 years ago, Linda Sobell and colleagues attempted to implement an intervention informed by studies of self-change in the community^12^ and did not find meaningful differences between brief, community-level interventions and just handing out pamphlets. Whether it is possible to developing effective interventions based on self-change studies remains an open question.

However, two important take-home messages from the article by Day et al remain. First, as the authors suggest, shedding light on the fact that resolution is not only possible but indeed very common may reduce the stigma associated with AOD problems.^13^ People with substance use disorders experience and internalise stigma, including during interactions with healthcare providers, which may prevent them from seeking treatment.^14^ Knowing that it is possible to get out on the other side is important and can boost hope. The second important message is that the notion that some people change without help should be understood in the right context. There is a vast difference between simple normative changes in substance use due to changing life circumstances, such as marriage or becoming a parent, and increasing maturity on the one hand^15^ and resolution of substance use disorders among people with complex and multifaceted problems on the other. To shed some light on the issue of changing life circumstances as an explanation for resolving an AOD problem, the group behind this study could analyse the age at which their respondents resolved their AOD problem in relation to assisted or unassisted resolution.

The fact that a large proportion of people change on their own does not mean that treatment services are not needed. The people who do change on their own have fewer and less complex problems than those who use services. Those who seek treatment are those who may have tried on their own but failed.^2^ If anything, services should be tailored to those who have more severe or co-occurring problems – people with early-onset substance use, a history of mental health problems, and a history of criminal justice involvement – for the sake of people with AOD problems, but also for the sake of their families and significant others.

Future studies could include settings that are much more diverse than the UK and the USA, including low- and middle-income countries. In low- and middle-income countries, very different healthcare systems, community-based treatment services, cultural views on AOD use, prevalence and patterns of AOD use, and poverty may all influence what it means to identify and resolve an AOD problem.

In conclusion, Day and colleagues found small but important differences in how people resolved AOD problems in the UK compared with the USA. This study provides ways forward for the field of recovery from AOD problems, both assisted and unassisted.
